# Active learning through journal clubs in pharmacy education: assessment of student experiences, outcomes, and attitudes

**DOI:** 10.3389/fmed.2026.1755033

**Published:** 2026-02-23

**Authors:** Malaz J. Gazzaz, Saad M. Wali

**Affiliations:** 1 Pharmaceutical Practices Department, College of Pharmacy, Umm Al-Qura University, Makkah, Saudi Arabia; 2 Pharmacology and Toxicology Department, College of Pharmacy, Umm Al-Qura University, Makkah, Saudi Arabia

**Keywords:** active learning, critical thinking, Evidence-Based Medicine, journal club, pharmacy education, pharmacy students, Saudi Arabia, Student engagement

## Abstract

**Introduction:**

Journal clubs are established pedagogical tools that enhance pharmacy students' critical appraisal and evidence-based practice skills. However, few studies have examined longitudinal changes following repeated exposure to such activities. This study assessed the outcomes, perceptions, and engagement of pharmacy students after two sequential journal club experiences within an Evidence-Based Medicine course.

**Methods:**

A cross-sectional comparative study was conducted among fifth-year undergraduate pharmacy students at Umm Al-Qura University, Makkah, Saudi Arabia. The second journal club experience occurred 3 months after the first one and used the same validated survey to measure three domains: learning outcomes, perceptions and attitudes, and feedback and future improvements. Descriptive statistics, Wilcoxon rank-sum, and chi-squared/Fisher's exact tests were used to compare scores between cycles, with significance set at *p* < 0.05.

**Results:**

seventy seven students participated in each experience. Reliability improved across all domains (learning outcomes α = 0.666 → 0.774; perceptions α= 0.782 → 0.813; feedback α= 0.647 → 0.710). Significant gains were observed in critical thinking (77.9% vs. 58.4%, *p* = 0.009) and data analysis (59.7% vs. 42.9%, *p* = 0.036). However, satisfaction and willingness to participate again declined (68.8% vs. 84.4%, *p* = 0.022), and mean feedback scores decreased (3.6 ± 1.5 vs. 2.7 ± 2.2, *p* = 0.005). No significant differences were found in overall learning outcome or perception scores.

**Conclusion:**

Repeated exposure to journal club activities enhanced students' analytical and critical thinking skills but reduced their satisfaction and enthusiasm for future participation. While the intervention strengthened cognitive learning domains, declining affective engagement suggests the need for curricular adjustments such as varied formats, reflective components, and diversified facilitation to sustain motivation and optimize educational impact in longitudinal learning designs.

## 1 Introduction

In health professions education, journal clubs are established pedagogical tools designed to enhance students' competencies in evidence-based practice, including critical appraisal, research literacy, and knowledge translation. Within pharmacy education, these interventions serve to bridge theoretical instruction and clinical reasoning by engaging students in active examination of research methodology, biases, statistical outcomes, and their implications for patient care (1, 2). A recent scoping review examining journal and book clubs in pharmacy education found that 86% of included studies focused on journal clubs, primarily used to teach evidence-based practice, drug literature evaluation, and biostatistics; however, the review highlighted important methodological limitations, noting that most studies relied on cross-sectional designs and student perceptions, with insufficient evidence on longitudinal skill development and higher-order outcomes such as interprofessional collaboration (3).

The theoretical basis for journal club activities lies in experiential learning and constructivist educational frameworks. According to Kolb's experiential learning cycle, learners transition through concrete experience, reflective observation, abstract conceptualization, and active experimentation (4–6). In a journal club, students engage with authentic research (concrete experience), critique and discuss the findings (reflective observation), derive general methodological principles (abstract conceptualization), and consider application in their future practice (active experimentation). Concurrently, constructivist theory posits that learners construct knowledge through social interaction and active inquiry rather than passive reception of information (7). Journal clubs thus function as social learning forums where students collaboratively interrogate research and co-construct meaning, reinforcing higher-order reasoning and professional identity formation.

Despite their widespread adoption in pharmacy curricula, evidence evaluating journal club effectiveness remains fragmented. Existing studies are predominantly cross-sectional, emphasize short-term perceptions rather than longitudinal outcomes, and rarely examine the effects of repeated exposure to journal club activities over time. Consequently, it remains unclear whether successive journal club cycles lead to sustained improvement in learning outcomes, diminishing returns, or potential learner fatigue—an issue increasingly recognized in higher education where repetitive active-learning strategies may reduce motivation despite cognitive gains (3, 8–12).

In pharmacy programs, one recent study of a structured journal club embedded in an evidence-based medicine course reported that 89.4% of students acquired new knowledge and 77.7% gained greater confidence in critically evaluating research, with significantly higher outcomes among more advanced students (13). While these findings support the educational value of journal clubs, the study did not assess within-cohort change over time or evaluate how repeated journal club exposure influences student perceptions, engagement, or measurement reliability, thereby limiting conclusions regarding longitudinal impact.

Addressing these gaps, the current study reports on a second iteration of a journal club activity delivered to the same pharmacy cohort 3 months after the first cycle, utilizing a previously validated survey instrument and comparable implementation approach. Guided by experiential and constructivist learning theories, this research aims to examine whether repeated engagement with journal club tasks improves student outcomes (learning, perceptions, attitudes) and retains or improves internal reliability of measurement. Accordingly, the study compares domain scores across cycles, evaluates Cronbach's alpha for each domain, and interprets findings in the context of cognitive skill acquisition and learner engagement over time.

## 2 Methods

### 2.1 Study setting and participants

This cross-sectional study was conducted to evaluate pharmacy students' experiences, learning outcomes, perceptions, and feedback regarding their participation in a Journal Club activity across two separate settings, with a three-month interval between them to align with the course timetable and academic calendar. This interval was selected to allow students adequate time to consolidate feedback and learning from the initial experience while maintaining comparable teaching conditions and minimizing confounding changes in course delivery. The study included male undergraduate pharmacy students in their 5th-year at Umm Al-Qura University, Makkah, Saudi Arabia.

The first experience served as a baseline assessment, which was detailed in a previous study by Almuzaini et al. (13), involving a cohort of 207 eligible male 5th-year pharmacy students enrolled in the Evidence-Based Medicine (EBM) course. For the current study (second experience), a convenience sample of students from the same cohort was included. All male 5th-year students who participated in the second Journal Club activity, completed the required course components, and consented to participate were eligible for inclusion.

Students were excluded if they did not attend the second Journal Club session, failed to complete the post-activity questionnaire, or declined participation. As a result, a total of 77 students constituted the final sample for the second study. No additional randomization or stratification procedures were applied.

### 2.2 Journal club activity and data collection

This Journal Club activity was conducted as part of the Evidence-Based Medicine (EBM) course assessment, contributing 25% of the total course grade. Each group was required to select and present a recent phase 3 or 4 randomized controlled trial (RCT). The presentations were evaluated by two academic judges based on the students' comprehension and detailed understanding of the study and following an evaluation rubric. Students were divided into groups of 4–5 members and were provided with structured guidelines and rubrics to ensure consistency in article selection, analysis, and presentations. Each session concluded with interactive discussions to facilitate peer learning and instructor feedback. A previously structured and validated survey developed by Almuzaini et al. (13) was re-administered immediately after the Journal Club sessions to assess students' engagement, learning outcomes, perceptions, and feedback.

### 2.3 Scoring for domains

The learning outcomes domain comprised five items assessing improvements in knowledge, skills, and confidence following the journal club activity. Four items were binary (Yes/No) and scored dichotomously (No = 0, Yes = 1), covering perceived gains in knowledge acquisition and selected skills (e.g., academic writing, teamwork, and critical thinking). One item assessed confidence in evaluating research and was rated on a 5-point Likert-type scale ranging from 1 (Not at all confident) to 5 (Much more confident). The learning outcomes score was calculated by summing the five item scores, with higher scores indicating more favorable learning outcomes. All items were equally weighted, and the multiple-choice item was coded as a single binary item to avoid disproportionate influence on the total score.

The Perception and Attitude domain included three items assessing students' views on the usefulness and impact of the journal club activity. Responses regarding whether the skills learned would be useful for future courses or careers were scored on a 5-point Likert scale, ranging from −2 (Strongly disagree) to 2 (Strongly agree). Changes in perception regarding the importance of staying updated with current research were scored on a 3-point scale: 0 (No change), 1 (Yes, somewhat), and 2 (Yes, significantly). The likelihood of recommending the activity to others was rated on a 5-point Likert scale, ranging from −2 (Very unlikely) to 2 (Very likely). The total score for this domain was calculated by summing the scores of all three items, with possible values ranging from −4 to 6.

The Feedback and Future Improvements domain also contained three items assessing the applicability of the skills gained, willingness to participate in similar future activities, and overall satisfaction with the journal club. These items were scored similarly, except for willingness to participate, which was scored dichotomously (No = 0, Yes = 1). Total scores for this domain were derived by summing all item scores, with possible values ranging from −4 to 4.

### 2.4 Ethics approval and consent to participate

A review and approval for this research were granted by the Scientific Biomedical Research Ethics Committee at the College of Medicine, Umm Al-Qura University's Institutional Review Board (IRB) (Approval Number: HAPO-02-K-012-2024-12-2424), and the study was performed according to the Declaration of Helsinki and relevant national laws on research ethics. Giving the questionnaire was voluntary, and participants gave their digital consent before starting. Participants were told through the form that the study sought to find out about workplace experiences and that their data would be kept anonymous.

No personal information, such as names, student IDs or IP addresses, was recorded or investigated in the study. Responses were taken through a private, encrypted survey platform that the university runs through its IT resources. Participants were not given any incentives, so they could drop out of the survey whenever they wanted, and there would be no penalty for doing so.

### 2.5 Statistical analysis

Descriptive statistics were used to summarize numerical scores and categorical responses. Differences in numerical scores between the first and second experiences were assessed using the Wilcoxon rank sum test. Comparisons of categorical variables between the two experiences were conducted using Pearson's chi-squared test or Fisher's exact test, as appropriate. Using independent tests was prioritized over paired tests in the current study since paired data could not be identified or matched, an approach which was previously suggested (14). Statistical significance was set at *p* < 0.05. All analyses were conducted using RStudio (version 2024.9.1.394, Boston, MA, USA) with R version 4.4.2.

## 3 Results

### 3.1 Numerical scores and reliability analysis of domains

The reliability analysis, measured using Cronbach's alpha, indicated acceptable internal consistency across domains. The learning outcomes domain showed moderate reliability in the first experience (α = 0.666), which improved in the second experience (α = 0.774). The perceptions and attitudes domain demonstrated strong reliability, increasing from 0.782 in the first experience to 0.813 in the second. The feedback and future improvements domain exhibited lower reliability initially (α = 0.647) but improved in the second experience (α = 0.710). The number of items assessed within each domain remained consistent, with 10 items for learning outcomes and three items each for perceptions and attitudes, as well as feedback and future improvements (Table 1).

**Table 1:** Description of the numerical scores and reliability analysis of domains in the current study.

**Characteristic**	**Median (Q1–Q3)**	**Mean ±SD**	**Min–max**	**Cronbach alpha**	**No. of items**
Learning outcomes (first experience)	8.0 (7.0–10.0)	8.4 ± 2.9	−3.0 to 13.0	0.666	10
Learning outcomes (second experience)	8.0 (6.0–11.0)	8.0 ± 4.1	−5.0 to 13.0	0.774	10
Perceptions and attitudes (first experience)	4.0 (3.0–6.0)	4.2 ± 1.9	−2.0 to 6.0	0.782	3
Perceptions and attitudes (second experience)	4.0 (2.0–6.0)	3.4 ± 2.7	−4.0 to 6.0	0.813	3
Feedback and future improvements (first experience)	4.0 (3.0–5.0)	3.6 ± 1.5	−2.0 to 5.0	0.647	3
Feedback and future improvements (second experience)	3.0 (2.0–5.0)	2.7 ± 2.2	−4.0 to 5.0	0.710	3

### 3.2 Differences in numerical scores across experiences

There was a significant decrease in feedback and future improvement scores between the first and second experiences (3.6 ± 1.5 vs. 2.7 ± 2.2, *p* = 0.005). However, no significant differences were observed in learning outcomes (*p* = 0.962) or perceptions and attitudes (*p* = 0.093, Table 2).

**Table 2:** Statistical differences in the numerical scores of different domains.

**Characteristic**	**First experience *N* = 77**	**Second experience *N* = 77**	***p*-value**
Learning outcomes	8.4 ± 2.9	8.0 ± 4.1	0.962
Perception and attitude	4.2 ± 1.9	3.4 ± 2.7	0.093
Feedback and future improvements	3.6 ± 1.5	2.7 ± 2.2	0.005
Mean ± SD			
Wilcoxon rank sum test			

### 3.3 General experience of students

A significantly higher proportion of students in the second experience found the journal club “not engaging at all” compared to the first experience (9.1% vs. 0.0%, *p* = 0.009). However, the majority in both experiences rated the activity as “very engaging” (48.1% in the first experience vs. 51.9% in the second experience). No significant differences were observed in students' perceptions of working in pairs (*p* = 0.437, Table 3).

**Table 3:** General experience of students regarding first and second experiences.

**Characteristic**	**First experience *N* = 77**	**Second experience *N* = 77**	***p*-value**
Did you find the idea of the journal club activity engaging?			0.009
Not engaging at all	0 (0.0%)	7 (9.1%)	
Not very engaging	8 (10.4%)	2 (2.6%)	
Neutral	0 (0.0%)	0 (0.0%)	
Somewhat engaging	32 (41.6%)	28 (36.4%)	
Very engaging	37 (48.1%)	40 (51.9%)	
Did you find working in pairs beneficial to your learning experience?	58 (75.3%)	62 (80.5%)	0.437
If yes, how did working with a partner enhance your learning?^*^			0.097
Collaboration and teamwork	7 (20.0%)	20 (48.8%)	
Knowledge sharing and learning	17 (48.6%)	14 (34.1%)	
Problem solving and clarification	3 (8.6%)	2 (4.9%)	
Skill development	3 (8.6%)	3 (7.3%)	
Time efficiency and simplification	5 (14.3%)	2 (4.9%)	

The values are given as *n* (%).

Fisher's exact test; Pearson's Chi-squared test.

^*^The variable had 23 missing records in the First experience and 21 missing records in the second experience.

### 3.4 Learning outcomes

A significantly higher proportion of students in the second experience reported improvements in critical thinking (77.9% vs. 58.4%, *p* = 0.009) and data analysis (59.7% vs. 42.9%, *p* = 0.036) compared to the first experience. No significant differences were noted in acquiring new knowledge or skills (*p* > 0.999) or in confidence in critically evaluating research (*p* = 0.282). Additionally, fewer students in the second experience found the activity beneficial for literature research, whether beneficial (39.0% vs. 46.8%) or very helpful (42.9% vs. 49.4%, *p* = 0.023). No significant differences were noted in academic writing students' feeling of confidence in the critical evaluation of research (*p* = 0.282) or the perception that the journal club helped in applying theoretical knowledge to real-world research (*p* = 0.881, Table 4).

**Table 4:** Learning outcomes of students in the first and second experiences.

**Characteristic**	**First experience *N* = 77**	**Second experience *N* = 77**	***p*-value**
Did you acquire new knowledge or skills from participating in this activity?	69 (89.6%)	69 (89.6%)	>0.999
**Which specific skills do you feel were most improved through this activity?**			
Academic writing	33 (42.9%)	34 (44.2%)	0.871
Team collaboration	48 (62.3%)	43 (55.8%)	0.413
Critical thinking	45 (58.4%)	60 (77.9%)	0.009
Data analysis	33 (42.9%)	46 (59.7%)	0.036
Presentation skills	56 (72.7%)	50 (64.9%)	0.297
Literature search	49 (63.6%)	45 (58.4%)	0.509
Do you feel more confident in critically evaluating research after this activity?			0.282
Not at all confident	0 (0.0%)	3 (3.9%)	
No, not more confident	3 (3.9%)	5 (6.5%)	
Neutral	0 (0.0%)	0 (0.0%)	
Yes, slightly more confident	36 (46.8%)	30 (39.0%)	
Yes, much more confident	38 (49.4%)	39 (50.6%)	
How beneficial was the activity in helping you understand how to conduct a literature search?			0.023
Not beneficial at all	0 (0.0%)	4 (5.2%)	
Not very beneficial	2 (2.6%)	9 (11.7%)	
Neutral	0 (0.0%)	0 (0.0%)	
Beneficial	37 (48.1%)	31 (40.3%)	
Very beneficial	38 (49.4%)	33 (42.9%)	
How effectively do you think the Journal Club helped you in applying theoretical knowledge to real-world research?			0.881
Strongly disagree	3 (3.9%)	4 (5.2%)	
Disagree	4 (5.2%)	6 (7.8%)	
Neutral	0 (0.0%)	0 (0.0%)	
Agree	34 (44.2%)	31 (40.3%)	
Strongly agree	36 (46.8%)	36 (46.8%)	

The values are given as *n* (%).

Pearson's Chi-squared test; Fisher's exact test.

### 3.5 Perceptions, attitudes, and future suggestions

A significantly lower proportion of students in the second experience expressed willingness to participate in similar activities in the future (68.8% vs. 84.4%, *p* = 0.022). Overall satisfaction with the journal club was also lower in the second experience, with a significant increase in dissatisfaction (*p* = 0.009). When asked what they liked most about the activity, fewer students in the second experience mentioned presentation skills (3.0% vs. 15.6%, *p* = 0.011). More students in the second experience selected “nothing” as their favorite aspect of the activity (13.4% vs. 1.3%, *p* = 0.006). Regarding suggested improvements, a significantly higher proportion of students in the second experience indicated a need for changes to the activity structure and requirements (24.6% vs. 8.5%, *p* = 0.010). Additionally, fewer students in the second experience were satisfied with the current setup (21.7% vs. 49.3%, *p* < 0.001, Table 5).

**Table 5:** Perceptions, attitudes, and future suggestions as indicated by students in the first and second experiences.

**Characteristic**	**First experience *N* = 77**	**Second experience *N* = 77**	***p*-value**
Will the skills you learned during this activity be useful in your future courses or professional career?			0.356
Strongly disagree	1 (1.3%)	2 (2.6%)	
Disagree	2 (2.6%)	7 (9.1%)	
Neutral	0 (0.0%)	0 (0.0%)	
Agree	33 (42.9%)	32 (41.6%)	
Strongly agree	41 (53.2%)	36 (46.8%)	
Has participating in the Journal Club changed your perception of the importance of staying updated with current research?			0.266
No change	5 (6.5%)	10 (13.0%)	
Yes, somewhat	30 (39.0%)	33 (42.9%)	
Yes, significantly	42 (54.5%)	34 (44.2%)	
How likely are you to recommend this activity to other students?			0.063
Very unlikely	0 (0.0%)	6 (7.8%)	
Unlikely	8 (10.4%)	11 (14.3%)	
Neutral	0 (0.0%)	0 (0.0%)	
Likely	28 (36.4%)	26 (33.8%)	
Very likely	41 (53.2%)	34 (44.2%)	
Will the skills you learned in this activity be applicable in your future courses or professional life?			0.099
Strongly disagree	0 (0.0%)	2 (2.6%)	
Disagree	7 (9.1%)	8 (10.4%)	
Neutral	0 (0.0%)	0 (0.0%)	
Agree	28 (36.4%)	38 (49.4%)	
Strongly agree	42 (54.5%)	29 (37.7%)	
Would you like to participate in similar activities in the future?	65 (84.4%)	53 (68.8%)	0.022
Please rate your overall satisfaction with the journal club activity:			0.009
Very dissatisfied	0 (0.0%)	5 (6.5%)	
Dissatisfied	2 (2.6%)	7 (9.1%)	
Neutral	0 (0.0%)	0 (0.0%)	
Satisfied	37 (48.1%)	41 (53.2%)	
Very satisfied	38 (49.4%)	24 (31.2%)	
**What did you like most about the journal club activity?** ^*^			
Nothing	1 (1.3%)	9 (13.4%)	0.006
Engagement and enjoyment	8 (10.4%)	11 (16.4%)	0.286
Presentation skills	12 (15.6%)	2 (3.0%)	0.011
Research and literature search	26 (33.8%)	17 (25.4%)	0.272
Skill development and learning	10 (13.0%)	15 (22.4%)	0.137
Teamwork and collaboration	17 (22.1%)	9 (13.4%)	0.179
Other	3 (3.9%)	4 (6.0%)	0.705
**What would you change to improve this activity in the future?** ^#^			
Activity structure and requirements	6 (8.5%)	17 (24.6%)	0.010
Engagement and enjoyment	0 (0.0%)	2 (2.9%)	0.241
Satisfaction with current setup	35 (49.3%)	15 (21.7%)	<0.001
Skill development and learning	12 (16.9%)	15 (21.7%)	0.468
Team structure and participation	8 (11.3%)	2 (2.9%)	0.097
Time management	6 (8.5%)	8 (11.6%)	0.535
Other	4 (5.6%)	10 (14.5%)	0.081

The values are given as *n* (%).

Fisher's exact test; Pearson's Chi-squared test.

^*^The variable had 10 missing records (second experience).

^#^The variable had 14 missing records (six in the first experience and eight in the second experience).

## 4 Discussion

This study evaluated the second iteration of a structured journal club activity in a pharmacy undergraduate program, tracking changes in students' learning-outcome domains (critical thinking, data analysis, literature search), perceptions/attitudes (satisfaction, willingness to re-engage), and instrument reliability (Cronbach's α). The main findings were two-fold: (1) cognitive-skill domains, i.e., critical thinking and data analysis, showed statistically significant improvement after the second cycle; (2) affective/engagement domains, i.e., student satisfaction, feedback scores, and willingness to repeat the activity, declined. These outcomes warrant a nuanced interpretation in light of educational theory, the existing literature on journal clubs in healthcare education, and broader work on student engagement and active-learning fatigue.

### 4.1 Improvement in cognitive skills

The observed increase in critical thinking and data-analysis scores aligns well with prior literature on journal clubs as vehicles for enhancing research literacy and evidence-based practice capabilities in health-professional education. For example, journal clubs may enhance participants' ability to critically appraise literature and translate findings into practice, thereby helping to bridge the gap between theory and practice in clinical settings (15–17). Similarly, the recent study by Almuzaini et al. (13) in pharmacy education found high proportions of students reporting new knowledge acquisition (89.4 %) and increased confidence in critical evaluation (77.7 %) after one cycle. The current findings suggest that repeated exposure, in this case, a second cycle, consolidates those gains, particularly in analytical domains. From the vantage of experiential learning theory and constructivist frameworks, this makes sense: the first journal club provides concrete experience and reflective observation; the second cycle allows for abstract conceptualization and active experimentation (i.e., applying previous critique skills to new articles). Moreover, the improvement in Cronbach's α across domains suggests that the instrument used for measurement became more reliable in the second cycle, indicating greater internal consistency of students' responses. This may reflect increasing coherence in how students interpret items as their experience grows, another indicator of progressive maturation in the activity's impact.

### 4.2 Decline in affective/engagement domains

In contrast to the cognitive gains, the decrement in student satisfaction, feedback scores, and willingness to re-engage is notable and must be interpreted with care. While cognitive domains improved, the affective engagement declined. This divergence echoes broader concerns in higher education regarding learner fatigue and reduced motivation in repetitive active-learning tasks. For instance, Mamani-Benito et al. (18) found emotional exhaustion to be a negative predictor of academic satisfaction, whereas engagement remained a positive predictor. That is consistent with our finding: although students may have developed skills (cognitive engagement), their emotional and motivational engagement may have suffered.

In the context of journal clubs, there are plausible mechanisms. First, the novelty effect may wear off on repeated implementations. The first exposure may generate excitement, collaboration, novelty, and challenge; the second may feel more routine, less stimulating, or more burdensome. Second, if the second cycle replicates the same format and intensity without variation, students may perceive redundancy rather than progression, leading to lower satisfaction despite actual skill gain. Third, workload and cognitive load might increase: as students become more proficient, their critique tasks may demand more effort, potentially reducing the “fun” or perceived ease of the activity.

These considerations are consistent with active learning research showing that while structured, collaborative, and task-oriented models tend to yield better cognitive outcomes, they do not automatically sustain motivation or emotional engagement unless adapted over time (19, 20). Additionally, literature on student engagement emphasizes the importance of psychological support, scaffolding, and variation in task design to sustain motivation (19–21).

Importantly, the observed decline in satisfaction and willingness to re-engage should not be interpreted as evidence of pedagogical failure, but rather as a signal that sustained engagement in repeated active-learning tasks requires intentional variation and motivational redesign. Educational research suggests that repeated exposure to the same instructional format, even when cognitively effective, may lead to reduced affective engagement if novelty and perceived progression are not maintained. This distinction between learning effectiveness and learner satisfaction underscores the need to balance instructional rigor with adaptive engagement strategies.

### 4.3 Theoretical and curricular implications

The divergence observed between cognitive gains and declining affective engagement has important theoretical and curricular implications for the design of sequential active-learning interventions. Putting these findings into a theoretical context, the juxtaposition of skill improvement and declining satisfaction highlights that educational interventions must address both cognitive and affective domains simultaneously. From a constructivist and experiential learning perspective, the second cycle accomplished stronger abstract conceptualization and active experimentation (skill build), however, neglected the motivational scaffolding for affective engagement and self-regulation. Curricularly, this suggests that when implementing sequential or repeated journal club activities, pharmacy educators should anticipate diminishing novelty and proactively diversify tasks to maintain engagement. For example, subsequent cycles might vary article formats (qualitative vs. quantitative), include interdisciplinary collaboration, introduce peer-facilitation, or integrate real-world clinical impact reflections rather than purely methodological critique. The literature supports that varying formats and integrating practice relevance improve both engagement and outcomes (22–24).

From a measurement standpoint, improvement in Cronbach's α reinforces confidence in the instrument's longitudinal reliability, but the different trajectories for cognitive vs. affective domains caution that domain-specific interventions may be needed rather than a one-size-fits-all approach.

### 4.4 Strengths and limitations

The study's strengths include its repeated-cycle design—rare in pharmacy education—and the use of a validated survey instrument applied consistently across both cycles, allowing for reliable within-cohort comparisons of outcomes. However, as the evaluation included only two consecutive journal club cycles, the findings should be interpreted as short-term changes between two iterations rather than definitive evidence of longer-term trends or the “evolution” of impact over time. Additional cycles are needed to determine whether outcomes continue to improve, plateau, or decline with repeated exposure.

Despite these strengths, several limitations should be acknowledged. The absence of paired-data identifiers prevented true longitudinal tracking of individual students, meaning that the findings represent cohort-level trends rather than individual progress. As a result, individual-level trajectories of skill development or disengagement could not be examined, potentially obscuring within-student variability across journal club iterations.

The study also relied on self-reported perceptions rather than objective performance measures, such as appraisal or assessment scores, which may introduce response bias. Additionally, as the study was conducted within a single institution and cohort, the results may not be generalizable to other programs with different student demographics or curricular structures. Although a decline in engagement was observed, emotional exhaustion, workload, and burnout-related factors were not measured directly, limiting interpretation of the underlying causes.

Moreover, the study included only male students, as male and female pharmacy students at Umm Al-Qura University are taught on separate campuses with distinct schedules and facilities. This gender-based limitation may further restrict the generalizability of the findings. Future studies including mixed-gender and multi-institutional cohorts, incorporating objective performance indicators and multiple repeated cycles, are therefore essential to confirm the transferability and sustainability of these findings across diverse educational contexts.

### 4.5 Future research directions

Future studies should incorporate qualitative data (e.g., student narratives) to explore the underlying reasons for declining engagement, along with longitudinal tracking of individual students across multiple cycles. Including behavioral outcome measures, such as graded appraisal tasks or observed participation in journal clubs beyond the curriculum would also strengthen the evidence base. Further research should examine the impact of modifying journal club designs between iterations (e.g., peer-led vs. faculty-led formats or variation in article types and group structures) (12). Investigating the optimal number of iterations before diminishing returns in affective engagement occur, as well as whether mixed-mode formats (online or hybrid) can sustain motivation, would provide valuable insights for educational design. Additionally, future studies should adopt longitudinal approaches, include objective performance indicators, and involve both male and female students to enhance the validity and generalizability of findings.

## 5 Conclusion

In summary, this second-cycle implementation of a structured journal club within an undergraduate pharmacy curriculum demonstrated clear improvements in cognitive skill domains, including critical thinking and data analysis, but revealed a decline in student satisfaction and willingness to participate in future sessions. These findings highlight a dual challenge for educators: while repeated, structured active-learning interventions can enhance analytic competencies, sustaining affective engagement requires deliberate design adaptations, workload balance, and task variation. Moving forward, sequential journal club activities should integrate both cognitive scaffolding and motivational supports to maintain student engagement, foster lasting skill development, and maximize educational impact.

## Data Availability

The raw data supporting the conclusions of this article will be made available by the authors, without undue reservation.
